# Impacts of Micro(nano)plastics on Terrestrial Plants: Germination, Growth, and Litter

**DOI:** 10.3390/plants12203554

**Published:** 2023-10-12

**Authors:** Xiaodong Li, Rongyu Wang, Wei Dai, Yaning Luan, Jing Li

**Affiliations:** 1 The Key Laboratory for Silviculture and Conservation of Ministry of Education, College of Forestry, Beijing Forestry University, Beijing 100083, China; xiaodongli@bjfu.edu.cn (X.L.); bjfuwry@163.com (R.W.); daiwei1@bjfu.edu.cn (W.D.); 2 School of Environment and Civil Engineering, Jiangnan University, Wuxi 214122, China

**Keywords:** micro(nano)plastics, plants, toxicity, transport, germination

## Abstract

Micro(nano)plastics (MNP) are pervasive in various environmental media and pose a global environmental pollution issue, particularly in terrestrial ecosystems, where they exert a significant impact on plant growth and development. This paper builds upon prior research to analyze and consolidate the effects of MNP on soil properties, seed germination, plant growth, and litter decomposition. The objective is to elucidate the environmental behavior of MNP and their mechanisms of influence on the plant life cycle. The unique physicochemical and electrical properties of MNP enable them to modify soil structure, water retention capacity, and pH. They can potentially act as “electron shuttles” or disrupt natural “electron shuttles” in litter decomposition, thereby interfering with nutrient transport and availability in the soil. Furthermore, MNP can physically obstruct nutrient and water channels within plants, impacting nutrient and water absorption. Once infiltrating plant tissues, MNP can form eco-coronas with plant proteins. Together with MNP adsorbed on the plant’s surface and within its tissues, they disrupt normal physiological processes, leading to changes in photosynthesis, biomass, cellular toxicity, genetics, nutrient uptake, and gene expression. These changes, in turn, influence seed germination and plant growth and development. As a burgeoning research field, future studies should delve deeper into various aspects of these changes, such as elucidating the pathways and mechanisms through which MNP enter plant tissues, assessing their intensity and mechanisms of toxicity on different plant species, and exploring the relationship between micro(nano)plastics and “electron shuttles”. These endeavors will contribute to establishing a more comprehensive theoretical framework for understanding the environmental behavior of MNP and their impact on plants.

## 1. Introduction

**Figure 1:**
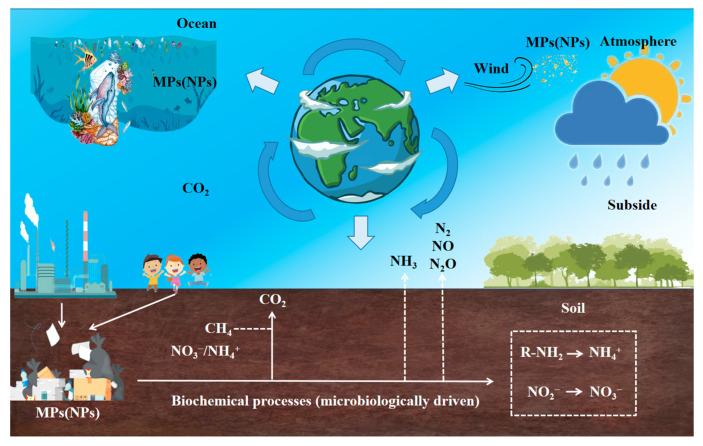
Transport and transfer of MNP between different ecosystems.

With the widespread use of plastic products, an increasing amount of plastic waste finds its way into the natural environment, where it undergoes physical, chemical, and biological weathering processes, ultimately breaking down into microplastics (MP, particles with diameters of <5 mm) and nanoplastics (NP, particles with diameters of <100 nm). These micro(nano)plastics (MNP) in the natural environment represent an emerging form of plastic pollution. They can be transported, transferred, and retained among different ecosystems under the influence of wind and water currents, exerting varying degrees of impact on ecosystem functionality (Figure 1). Recent research has demonstrated that MP and NP not only enter plant systems through their roots and leaves but also undergo internal translocation and accumulate within various plant tissues. This implies that MP and NP can enter the food chain, posing potential risks to human and animal health [1,2,3]. Consequently, micro(nano)plastic pollution is a significant challenge facing the world today [4].

The impact of MP and NP on the germination and growth development of plant seeds has consistently been a significant topic in related research fields. Since Rillig first addressed the adverse effects of accumulated MP in soil on soil properties and soil biodiversity, as well as the influence of MNP on plant and food safety [5,6,7], many researchers have separately undertaken studies on the effects of MNP on plants, considering both soil and plant perspectives. Previous research has revealed that, under the influence of physical retention and electrostatic forces, MNP can accumulate significantly and persistently in soil [8]. This not only makes soil a critical source and sink for MNP in other ecosystems but also means that, due to their unique chemical, physical, and electrical properties, MNP can alter soil characteristics and the direction and intensity of preexisting biochemical processes [9]. Primarily, the oxygen-containing functional groups on the surface of MP can weaken their aggregation capacity between soil particles, disrupting the stability of aggregates [10]. When MP are embedded into aggregates, they bind with aggregates at different particle levels, not only reducing their bulk density but also lacking sufficient cohesive or cementing functions, preventing the formation of stable aggregates [11]. Meanwhile, the large surface tension of MP gives them strong adsorption capabilities, enabling the accumulation of significant amounts of organic matter and microorganisms. This makes them a focal point for the decomposition and transformation of soil organic matter [12]. These alterations encompass interactions between particles, bio-degradation, dissolution, and adsorption, as well as changes and decomposition in the quality of litter material, all of which impact the composition of terrestrial ecosystems, altering their services and functions [13]. Consequently, this has direct or indirect consequences for the growth and developmental processes of plants [14]. However, many scholars often conduct indoor cultivation experiments to investigate the direct effects of MNP on plant growth. They have found that the aggregation and absorption of MNP on the root and leaf surfaces, as well as their processes of transport and transformation within the plant, can disrupt normal physiological processes. This disruption can induce changes in photosynthesis, biomass, cellular toxicity, genetics, nutrient absorption, and gene expression, all of which exert a profound impact on plants [15,16].

So far, all research findings have consistently indicated that MP and NP possess potential and far-reaching biological effects on plant development [17,18]. However, due to the relatively new nature of the related research, limited experimental methods, and the complexity of the impact of MNP on plants, most current studies are in the exploratory stage. Due to the lack of data support, some conclusions can only be based on theoretical inferences, and there have even been instances of conflicting findings. To facilitate further research on the influence of MNP on terrestrial plants and to elucidate the environmental behaviors of MNP and their mechanisms affecting the entire plant life cycle, this paper provides a comprehensive review of the impact of MNP on plants, delving into the potential effects of MNP on plant life. We particularly focus on the following aspects: (1) the impact of MNP on seed germination; (2) the effects of MNP on plant growth; (3) the influence of MNP on litter decomposition; and (4) the challenges faced and future research priorities.

## 2. The Impact of MNP on Plant Seed Germination

**Figure 2:**
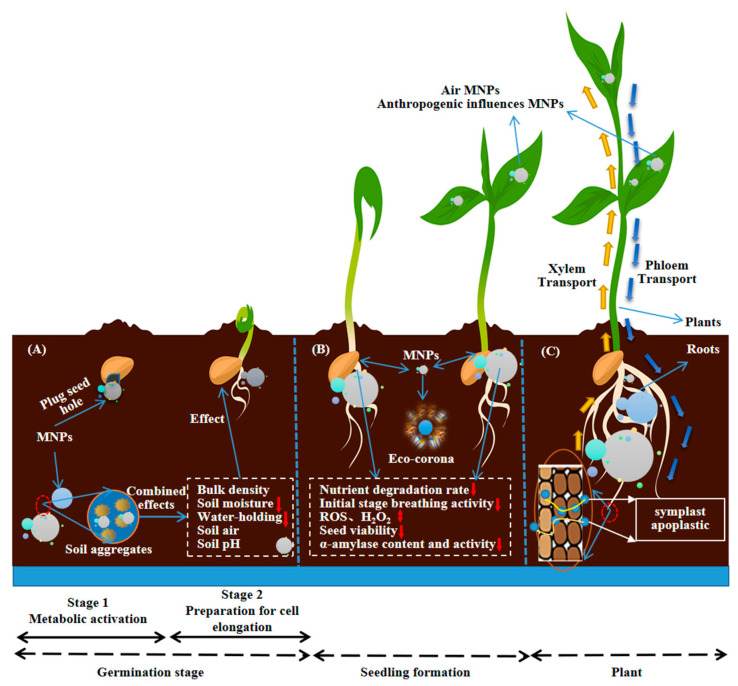
Effects of MNP on seed germination: (**A**) MNP affect external ecological conditions of the seed; (**B**) MNP effect internal physiological conditions of the seed; (**C**) plants take up MNP with the roots or root hairs or leaves as the main entry points to get MNP into plant tissues. Note: The red arrow indicates that the indicator goes up or down; the red circles represent local magnification.

MNP that enter the environment can alter the external soil conditions for seed germination (Figure 2A). This is primarily manifested in the following ways: Firstly, MNP can disrupt soil structure, reduce soil water retention capacity, and impact seed water absorption. Additionally, high concentrations of MP, acting as foreign components in the soil, exacerbate the aforementioned effects. Studies have shown that the accumulation of high concentrations of polyethylene microplastics (PE-MP) significantly affects soil water retention capacity and water retention characteristic curves, limiting the absorption of water by seed cells, leading to the inability of seed cell protoplasmic colloids to transition from a gel to a sol state [11].

Secondly, based on computed tomography (CT) scanning and soil pore three-dimensional reconstruction technology, it was discovered that adding PE-MP (2%, 950 μm) to sandy soil would decrease soil porosity and circularity, impacting the exchange of air within the soil. This, in turn, affects the supply of oxygen required for seed respiration and is unfavorable for seed germination [19]. Thirdly, MNP may potentially induce soil acidification, resulting in adverse effects on seed germination. Research indicates that plastic carbon dots (CDs), NP with a diameter smaller than 10 nm synthesized from non-biodegradable plastic waste, can function as nano-seed germination inducers. However, when the concentration of CDs exceeds a certain threshold, it leads to a decrease in soil pH, causing erosion on the surface of pea seed coats [20]. While this “erosion effect” has positive effects on seed coat expansion, softening, water absorption, and seed gas exchange, the persistent adhesion of MNP to the seed surface, owing to their small size and strong adsorption capacity, obstructs the nutrient and water uptake channels in seeds. This is detrimental to normal seed germination, offsetting the positive impact of the “erosion effect”. Further observation of pea seed sections revealed the presence of nano-clusters within seed tissues, confirming that CDs can adsorb onto the seed surface, further internalize, and accumulate within the seed coat, significantly affecting nutrient and water absorption by seeds [20,21]. Importantly, the negatively charged cell membranes and cell walls make positively charged MP more predisposed to adsorb and aggregate near plant tissues [21].

MNP also exert significant effects on the internal physiological conditions of germinating seeds (Figure 2B), primarily manifesting in their ability to influence the quantity and degradation rate of internal nutrients. During seed germination, cotyledons or endosperm storage organs serve as nutrient sources for the growth organs such as the embryonic root and shoot, establishing a “source-sink” relationship. However, results from optical coherence tomography (OCT) visualization studies show that PE-MP can hinder the initial internal activities of seeds, disrupting the normal supply of nutrients [21]. Furthermore, MNP also affect seed germination by interfering with seed physiological processes. The use of NP synthesized from non-biodegradable plastic waste (<10 nm) as inducers for pea seeds may induce an excessive production of reactive oxygen species (ROS) within the embryo, thereby inhibiting seed germination [20]. Additionally, hydrogen peroxide (H_2_O_2_) is an important internal stress signal molecule in seeds, and the presence of MP can affect the regulation of H_2_O_2_ during seed germination. With increasing concentrations of polystyrene microplastics (PS-MP), the H_2_O_2_ content in plant seeds exhibits a characteristic pattern of initially increasing and then decreasing. This suggests that under PS-MP stress, seeds can convert high levels of O_2_•^−^ present in the embryo into H_2_O_2_ through dismutation. It also indicates that different concentrations of PS-MP have differential effects on the physiological processes of seed germination [20,22,23].

Research has also found that hazardous monomers and various toxic substances contained within MNP are released in the form of leachate and absorbed by seeds, thereby affecting the conformation of α-amylase in seeds, delaying embryo growth and the emergence of the embryonic root [24]. Subsequently, multispectral analysis and computer simulations further confirmed that 100 nm PS can alter the activity of α-amylase and the structure of proteins [25]. While the results of both of these studies are consistent, some research suggests that changes in protein activity and function, as well as alterations in the toxicity and targeting ability of nanoparticles, may be due to the formation of eco-coronas [26]. The latest research has discovered that during seed germination, the interaction between MNP and seed cells results in the formation of eco-coronas, which further regulate the biological toxicity of nanoplastics on seed germination [27]. When humic acid (HA) and fluorescent polystyrene nanoparticles (Flu-PS) form an eco-corona, it affects the environmental behavior of the nanoparticles. Microscopic images show fewer healthy cells on the surface of tomato seeds in the Flu-PS-treated group, while the HA-treated group and Flu-PS+HA-treated group have more healthy cells on the seed surface, indicating that the formation of an eco-corona reduces the toxic effects of Flu-PS on seed germination [28]. It can be inferred that the formation of an eco-corona alters the surface properties of the original nanoparticles, reducing their physiological toxicity to seeds. Furthermore, as the eco-corona develops, particle size continues to increase (Figure 2B), ultimately preventing MNP from entering the interior of seeds, further reducing the extent of their negative impact on seed germination [26,28]. However, to date, there is a significant lack of research on the formation of eco-coronas and their effects on seed germination. The theoretical basis and data support for the research findings are insufficient, and further in-depth studies are urgently needed in this area.

## 3. Effects of MNP on Plant Growth

### 3.1. Impact on Plant Photosynthesis

MNP that enter the plant through organs such as the roots and leaves can undergo long-distance transport within the plant along the xylem under the influence of the transpiration flow, leading to internal redistribution. After exposing lettuce leaves to five types of MP, confocal laser scanning microscopy and scanning electron microscopy images confirmed the presence of such transport processes within lettuce plants [29]. It is believed that this transport and redistribution may affect plant photosynthesis [3,30]. Subsequent research has also shown that PS-MP with particle sizes ranging from 100 to 700 nm can be transported from carrot stems to different plant parts such as leaves, flowers, and fruits, and it has been clarified that the transport and redistribution processes within the plant can have multiple impacts on plant photosynthesis [31]. We note that the impact of MNP on plants is subject to the combined influence of various factors, including the type of MNP, environmental conditions, the accumulation and transmission of MNP within plant tissues, as well as differences among plant species (Table 1). The complexity of these combined factors results in some uncertainty regarding the precise effects of MNP on plant photosynthesis. Currently, it is speculated that MNP may impact plant photosynthesis due to several reasons. One of the primary reasons is that MNP may directly interfere with the charge separation and photochemical reactions of the photosynthetic reaction centers by reducing the electron transfer rate, promoting the accumulation of electrons in the plant’s photosynthetic reaction centers, and increasing ROS levels [18]. Secondly, the addition of MNP may reduce carbon assimilation in photosynthesis, affecting plant photosynthesis. The reduction in carbon assimilation can also inhibit carbon transfer in plant roots and soil, leading to nutrient deficiency and weakened root growth, affecting the distribution of trace and macro elements within the plant and slowing down leaf growth and photosynthetic efficiency, further exacerbating the aforementioned photoinhibition [32,33]. Additionally, MNP may affect the structure of water-soluble chlorophyll proteins, leading to changes in chlorophyll content and other parameters. Studies have confirmed that MNP can reduce the chlorophyll content in ryegrass, disrupting the balance of plant chlorophyll [7]; even under conditions of low nutrient levels, MNP can still interfere with normal chlorophyll content [34]. The electrical properties and environmental conditions of MNP are also important factors affecting photosynthesis. Different electrical properties of MNP have varying inhibitory effects on plant photosynthesis. Research indicates that positively charged MNP have a stronger inhibitory effect on plant photosynthesis compared to negatively charged ones [35]. Furthermore, complexes formed by the interaction of functional groups on the surface of MNP with organic pollutants and heavy metals in the soil may alter the bioavailability of organic pollutants and heavy metals, leading to a significant reduction in chlorophyll content in plants and affecting the normal progress of plant photosynthesis [36].

### 3.2. Impact on Plant Biomass

MNP demonstrates an impact on plant growth characteristics, biomass, root structure, and physiological–biochemical features, emphasizing the multi-faceted nature of the research (Table 2). These findings also indicate a general trend, namely, that MNP may potentially exert adverse effects on plants. Numerous studies have confirmed that MNP have a significant impact on plant biomass. However, there are significant differences in the effects on different plant species and organs, reflecting the complexity of these impacts. Plants such as onions and lettuce exposed to MNP show a significant reduction in belowground biomass (root biomass) and aboveground biomass, while in ryegrass they exhibit a stimulating effect on belowground biomass growth [42,43,44]. Additionally, different plant organs respond differently to MNP. When tomatoes are exposed to MNP, both root length and fresh weight decrease, with PS having a more pronounced impact on root length, whereas PE has a greater influence on root weight. This indicates that the type of MNP can also affect their impact on plant root systems [45,46]. The inconsistency in these results may be attributed to the interaction of factors such as plant physiological characteristics, rhizosphere environmental conditions, and the type and concentration of MNP.

Plant roots or root hairs constitute the main components of belowground biomass in plants and are also the primary organs interacting with MNP. The disruption of plant root ecology by MP is likely the primary reason for the reduction in belowground biomass. Root secretions and exudates have the capacity not only to capture and accumulate nano materials but also to absorb MNP dispersed in water, adsorbed on soil particles, and present in sediments [47]. This promotes the significant accumulation of MNP on the root surface, leading to the damage and disruption of root cells, which, in turn, affects further root development and growth [48]. Moreover, MP in the soil can serve as an additional carbon source or nutrient, stimulating soil microbial activity and exacerbating nutrient competition between plants and microbes, thereby affecting plant biomass [43,45].

The dual effects of damage by subterranean herbivores and mechanical injury can lead to the formation of large cracks in plant roots [3,49], facilitating the penetration of MNP larger than cell wall pores into plant living cells, resulting in intracellular migration and redistribution. This triggers physiological responses in plants, negatively impacting plant growth and development [31,50]. Research indicates that when plants are subjected to external stress, they generate a series of physiological responses characterized by the accumulation of reactive oxygen species, activation of antioxidant enzymes, and alterations in metabolic pathways. These physiological stress responses are essential factors influencing the rate of plant growth and biomass accumulation [37,38,39,51].

**Table 2:** Effect of MNP on plant biomass.

Test Object	MNP			Exposure Time	Effect	Mechanism	References
	Type	Size	Concentration				
strawberry	HDPE	2–5 mm	0.2 g/kg	5 m	HDPE affects plant height, stem thickness, biomass, root volume, and surface area.	The synthesis of chlorophyll and its stable binding with proteins is inhibited.	[52]
corn	PMFs	2.87 mm	0.5%	5 m	PMFs lead to a reduction in plant nitrogen uptake and biomass production.	Soil macro- and micro-porosity are influenced by PMFs.	[53]
plant community	EPS	200 μm	0.1% 0.2%	6 m	When EPS concentration is high, the total biomass and root biomass of the plant community are significantly lower than the control.	Exacerbated the generation of ROS and induced genetic toxicity in cells.	[54]
sweet potato	PVC	6.5 μm	100 mg/L 200 mg/L	14 m	The combined application with Cr(VI) reduces plant height, per plant fresh biomass, and chlorophyll content, among others.	MNP enhance the accumulation of Cr(VI) and its toxic effects on the physiological and biochemical characteristics of sweet potato plants.	[55]
wheat	PS	100 nm	0.01–10 mg/L	21 m	PS causes a reduction in the stem-to-root biomass ratio (S:R) in wheat seedlings.	Plants experiencing low nutrient supply exhibit an increased allocation to the root system.	[56]

Note: HDPE, high-density polyethylene; PMFs, polyester microfibers; EPS, expanded polystyrene; PVC, polyvinyl chloride.

### 3.3. Impact on Plant Antioxidant Characteristics and Cellular Toxicity

MNP, due to their high cellular affinity and large surface area, easily enter organisms and jointly induce bio-toxicity at the cellular and molecular levels, affecting the antioxidant characteristics of plants. The oxidative damage-induced oxidative stress response in plants is a major mechanism of MNP-induced ecotoxicology. Under MNP stress, plants transport MNP through the apoplastic pathway, leading to the accumulation of a large number of MNP in the intercellular regions of the roots and their movement towards the endodermis under osmotic pressure and capillary action [51,57]. This accumulation and transport process can result in the significant accumulation of reactive oxygen species (ROS), exacerbate lipid oxidation damage in plants, alter the activity of antioxidant enzymes, and lead to oxidative damage to cells [35,40], ultimately triggering the oxidative stress response in plants. Studies have shown that there is a linear relationship between the increase in MNP concentration and ROS accumulation in the aboveground parts of plants, with negatively charged MNP having a stronger stimulating effect on the activity of the antioxidant system. When high concentrations of PS and polypropylene (PP) are present, the resistance of tomato antioxidant enzymes and protein synthesis is inhibited, leading to the suppression of antioxidant enzyme activity in tomato cells and the accumulation of a large amount of ROS in the cells [46]. On the other hand, MNP may also affect the diffusion of ROS at the tissue and cellular levels, causing changes in cell biology and physiology, inhibiting the expression of plant antioxidant capabilities. Under MNP stress, the balance and diffusion of H_2_O_2_ in rice seedlings are affected, leading to localized membrane damage in plants. Further electron microscopy analysis confirms this, revealing that the generation of localized ROS leads to the degradation of rice seedling cell structures such as cell membranes and cell walls, preventing cells from exerting their antioxidant functions [58].

MNP often exhibit combined toxicity with heavy metals, affecting the activity of plant antioxidant enzymes. Studies have found that the combination of MP with Pb affects the activity of plant antioxidant enzymes. In this regard, the levels of the plant’s superoxide dismutase (SOD) increase with increasing Pb concentrations, while the presence of MP exacerbates the toxic effects of Pb on SOD, ultimately leading to an increase in ROS levels in the plant [59]. Similarly, the joint action of NP and Cd inhibits the activity of plant antioxidant enzymes by promoting the production and accumulation of ROS in the plant and the utilization of Cd by organisms, thus affecting plant growth [51]. Although these studies all indicate that MNP exhibit combined toxicity with heavy metals, it is currently unclear whether the involvement of other substances in the soil, given its complex ecological system, would further amplify the toxic effects of MNP.

At the cellular level in plant cells, MNP exhibit potential risks that may lead to cell death, dissolution, and the inhibition of cell growth, exerting direct toxic effects on cells. On the one hand, MNP affect the transport of nutrients within plant cells. In the symplast transport pathway of plants, nanoparticles may interact with membrane proteins, ion channel proteins, and aquaporin proteins, participating in the transport and internalization of substances through endocytosis, thus affecting the transport and absorption of other cellular nutrients, leading to impaired cell growth. Studies have found that in bright yellow2 (BY2) cells, PS-NP fluorescent beads can enter cells through both mesh protein-dependent and mesh protein-independent endocytosis, thereby affecting normal cell growth [60]. This not only confirms the symplast transport pathway of MP but is also consistent with previous research results on the endocytosis of MNP by plants. On the other hand, MNP affect plant cell mitosis. Changes in the mitotic index (MI) can reflect the severity of MNP cytotoxicity. According to research, under the influence of different concentrations of MNP, the MI of onions significantly decreases, and there is a significant difference in the percentage of MI at different stages of mitosis [61]. The decrease in MI may be due to the interference of MNP with the normal process of mitosis, leading to a reduction in the number of dividing cells and inhibiting mitosis [62]. Additionally, MNP can cause direct damage to the cell wall and cell membrane [63]. Due to the different zeta potentials of MNP, the stiffness of plant cell walls significantly decreases when plants are exposed to MNP, making the cells more fragile and susceptible to external damage [64]. Research has found that exposing both peanuts and lettuce roots to MNP can lead to the separation of plant cell cytoplasm from the cell wall, resulting in structural damage to plant cell walls and plasma membranes, affecting cell membrane permeability, disrupting the stable metabolic environment inside the cell, and thus exerting a toxic effect on cells [42,48].

### 3.4. The Impact on Plant Nutrient Uptake

Due to their small particle size and strong colloid stability, MNP persist in the environment and are easily transported, significantly affecting water and salt transport in soils and plant nutrient uptake. On the one hand, MNP affect soil moisture content and electrical conductivity, impeding the movement of soil moisture and the transport of salts across soil aggregates. Mainly due to the frequent electrification or generation of charges during the interaction between MP and natural organic matter, some negative charges in the soil are neutralized [65]. Moreover, the hydroxyl groups on the surface of MP can consume OH^-^ in the soil through deprotonation, leading to a reduction in the total amount of conductive substances in the soil, affecting soil electrical conductivity [66]. On the other hand, after the addition of MP, the contact angle of the soil increases. The soil particles not only exhibit hydrophobicity, but the MP also fill the pores of the soil particles, reducing the soil permeability coefficient and demonstrating a blocking effect that affects water infiltration. Simultaneously, as MP are hydrophobic materials themselves, they are not conducive to retaining soil moisture [67]. The combined effect of these two factors influences soil moisture content. This makes it difficult for nutrient ions in soil solutions to reach the surface of plant roots through mass flow and diffusion, and also hinders the release of nutrient ions adsorbed on solid soil particles into the liquid phase, affecting normal nutrient uptake by plants [68]. For example, Dong et al. found that the increase in residual plastic film in cotton fields affected soil quality, impeding the transport of soil moisture and nutrients, ultimately affecting the concentration of soil nutrients supplied to plants [68]. In addition, MNP can adhere to plant roots, blocking ion channels and cell wall gaps, and can also cause the heteroaggregation of particles with opposite charges in the gaps, leading to the disruption of root cell integrity [29,42]. For example, although the roots of rapeseed can directly contact the surface of soil particles, the presence of MNP with different charges obstructs the channels for nutrient absorption by rapeseed roots. This prevents the rapeseed roots from effectively capturing and absorbing nutrients, disrupting the uptake of essential nutrients by rapeseed. Additionally, it leads to an overall reduction in micronutrient elements such as iron, manganese, zinc, and copper, which is detrimental to the plant’s disease resistance [24]. Even at high concentrations of MNP, plant cells can suffer further damage, resulting in the inability of plants to absorb nutrients. According to research, high concentrations of PP and rubber particles can damage peanut plant root cells, preventing the normal absorption of nutrients by plants. This ultimately inhibits the plant’s uptake of nitrogen and nutrient growth, thereby disrupting soil nitrogen cycling [48].

Enzymes can catalyze nutrients and promote the absorption and utilization of nutrients by plants. However, the activity of plant enzymes is usually interfered with by MNP, thus affecting plant metabolism and nutrient absorption. For example, during plant respiration, MNP interfere with the enzymes involved in the release of energy from the oxidation of organic matter in plant cells; in the process of glucose metabolism, MNP can also interfere with the absorption and transport of plant nutrient elements by enzymes [31,69]. Furthermore, based on comprehensive metagenomics and metabolomics analyses, the same results were discovered. After the addition of MNP, the metabolism of three carbohydrates in plants (barley) and the activity of metabolic enzymes were significantly reduced, and the levels of soil metabolites also changed [48,70]. It can be observed that changes in enzyme activity lead to a reduction in plant metabolism, ultimately affecting the energy supply process for nutrient absorption by plants.

### 3.5. The Impact on Plant Gene Expression and Genetics

The regulation of gene expression in plants is a crucial process for maintaining life activities, but the presence of MNP disrupts this balance. Numerous studies have confirmed the significant effects of MNP on gene expression and genetics. However, there are substantial differences in the outcomes of these impacts on plants at different growth stages. Research has shown that under MNP stress, plants initiate various gene expression responses and induce adaptive stress signaling cascades across different growth stages and tissue types [71]. Specifically, during the vegetative stage, MNP primarily suppress the expression of genes encoding nitrate transporters in plant roots and genes involved in photosynthesis, inhibiting the nutritional growth of plants. In contrast, during the reproductive stage, genes encoding ammonium and nitrate transporters tend to be upregulated [72]. During the process of leaf photosynthesis, MNP inhibit the expression of genes associated with light harvesting, electron transport, and the photosystem, thereby affecting the expression of photosynthesis-related genes. For instance, in watercress, genes related to photosynthetic pigments such as *psbA* and *rbcL* are overexpressed under MNP stress, leading to the occurrence of genetic toxicity in watercress [73]. These findings collectively reflect the impact of MNP on the regulation of gene expression in plants.

Further research has revealed that MNP, in addition to affecting the regulation of plant gene expression, also exert direct toxic effects on cellular genetic stability. Upon entering plant cells, certain MNP can disrupt plant organelles and affect the functionality of the plant genome, leading to genetic toxicity. Because MNP directly damage plant cell DNA, they cause damage to an important regulatory factor, *cdc2*, which in turn induces genetic toxicity [62]. Furthermore, research has indicated that MNP can also induce chromosomal abnormalities and nuclear distortions. For example, MNP significantly reduce the onion’s chromosomal abnormality index and nuclear abnormality index, and induce the formation of micronucleated cells in onion root tip cells, leading to cytotoxicity and nuclear damage [74]. Additionally, MNP have an impact on chromosome constitution. Various types of abnormalities, such as micronucleated chromosomes, disjoined chromosomes, and multiple fragmented chromosomes, were observed in onion cells treated with MNP [61]. These abnormal changes lead to the stickiness, fragmentation, and fusion of chromatin and chromosomes, resulting in the formation of chromosomal bridges and irreversible alterations in chromosome constitution [75].

## 4. The Impact of MNP on the Decomposition of Plant Litter

**Figure 3:**
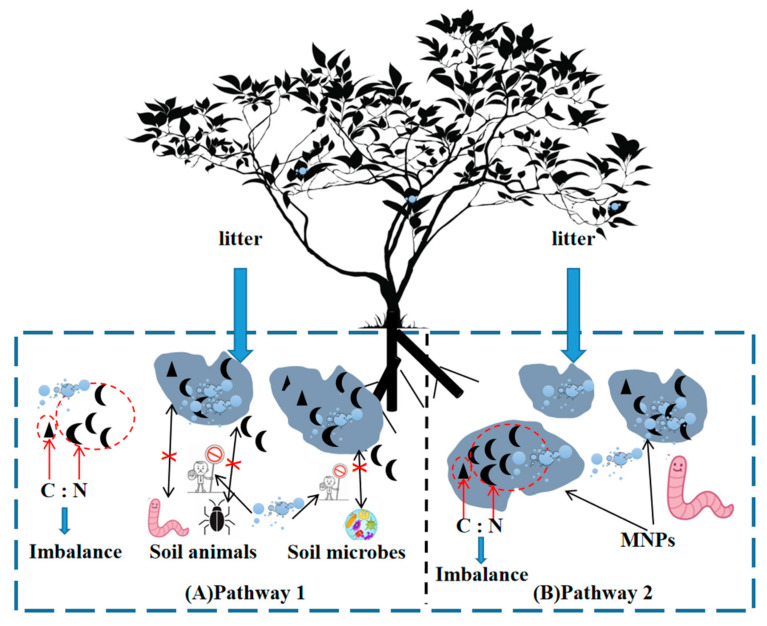
Two pathways through which MNP influence the decomposition of litter and fallen leaves: (**A**) MNP hinder the decomposition activity of microorganisms and soil fauna during the decomposition of plant litter and fallen leaves, affecting soil C:N; (**B**) The ‘dilution effect’ of MNP reduces the absorption of leaf nutrients, leading to imbalanced C:N in litter and changes in litter quality.

MNP have a profound impact on the quality of plant litter, and the mechanisms involved encompass several key aspects. Firstly, the effect is manifested in the ability of plant leaves to adsorb and absorb MNP. MNP are widely present in the atmosphere and possess the ability to be transported globally [76]. During atmospheric transport, the higher particulate capture capacity of plant leaves leads to the accumulation of a considerable quantity of MNP on leaf surfaces [1,77]. As plant leaf stomata are approximately 10μm in size, they are large enough to allow the entry of nanoscale plastic particles into the vascular system [3]. Moreover, the hydrophobic surface properties of MNP can enhance their affinity with leaves, allowing them to penetrate the leaf cuticle and its pores, thus accumulating in different parts of the plant and eventually returning to the soil as one of the components of litter, thereby altering the quality of the litter [8]. Furthermore, under electrostatic interactions, MNP exhibit different aggregation states on leaf surfaces. As adsorbents, they can become carriers for other pollutants, promoting the enrichment of volatile organic compounds or heavy metals on leaf surfaces, further affecting litter quality [77]. Secondly, the entry of MNP into the plant’s internal tissues can affect the nutrient effectiveness of the litter (Figure 3B). MNP that enter plant tissues are themselves organic carbon, with a carbon content as high as 80%. After plant senescence, this portion of organic carbon cannot be decomposed and returned to the soil as nutrients, leading to changes in the nutritional quality of the litter [1].

The decomposition of plant litter involves key steps such as the leaching of water-soluble compounds, fragmentation by soil organisms, and the material transformation carried out by microorganisms. Microorganisms play a crucial role in initiating and interrupting the material and energy cycling in the biosphere. Early research has found that MNP significantly affect the microbial decomposition process of litter (Figure 3A). Firstly, MNP directly slow down the microbial decomposition rate. Studies have shown that after the addition of MNP, the C:N of litter increases significantly [14]. The primary reason may be that the “dilution effect” of MNP, once they enter plant tissues, leads to a reduction in the nutrient content absorbed by plant leaves, resulting in an imbalance of litter C:N. This, in turn, dilutes the quantity of litter available to decomposers and hinders the decomposition rate [7]. Secondly, MNP alter the characteristics of existing microorganisms. Research indicates that MNP not only affect the diversity of endophytic bacterial communities in the root zone but also have a more significant impact on the changes in endophytic fungi [78,79]. The main reason is that MNP can input carbon sources into the soil for an extended period, and most of this carbon is in an inert state with slow degradation rates, leading to noticeable differences in soil C:N and changes in microbial activity and structure [6,14,80]. Studies have even found changes in soil microbial community diversity and structure after the application of ^13^C-labeled PE-MP [32]. Some research even suggests that during the seedling stage of barley plants, the bacterial network of the MNP treatment group exhibits higher vulnerability [78,79]. Thirdly, MNP impact soil organisms. Soil organisms also play essential ecological roles in the decomposition process, such as nematodes, which can ingest litter fragments and break them down into simple inorganic substances. However, the plant secondary metabolites derived from litter influenced by MNP may have potentially negative effects on soil nematodes, inhibiting their decomposition of litter [81].

The latest research results indicate that MNP may have an impact on the natural “electron shuttle” function during the decomposition of litter or even act as an “electron shuttle”, thereby influencing microbial decomposition activities. On the one hand, MP may also serve as “electron shuttles”, allowing microorganisms to form biofilms on their surfaces through attachment mechanisms, thereby acting as electron sinks or electron donors in microbial metabolism. This phenomenon plays a positive role in the migration and transformation of litter in the environment, thus positively affecting material cycling and energy flow in ecosystems [7,82]. Furthermore, due to the large specific surface area of MNP, they may interact with natural “electron shuttles”. In particular, the attachment of MP may create an interface between the electron shuttle and other materials or the environment, which could affect the interface properties and interactions of the electron shuttle, resulting in changes in the interactions between the electron shuttle and the surrounding environment [83]. Relevant research findings suggest that polystyrene microplastics (PS-MP) exhibit a high binding affinity for dissolved organic matter (natural “electron shuttles”), and oxygen-containing functional groups in MP are considered the optimal structures for binding with dissolved organic matter, influencing electron transfer during redox processes [84]. It is further inferred that after MNP adsorb onto the surface of natural “electron shuttles”, they may form complexes that affect the chemical structure of the natural “electron shuttles”, thereby influencing the electron transfer process. Further research may provide corresponding evidence to elucidate this process. Research has found that the photoreduction process of Ag^+^ involves the binding of Ag^+^ to weathered PS-MP carbonyl groups, and X-ray photoelectron spectroscopy and Fourier transform infrared spectroscopy have confirmed that carbonyl groups (including aldehydes) on weathered PS are key determinants for the reduction of Ag^+^, and an electron shuttle occurs between them [85]. However, there is currently a lack of related research to fill this research gap, and further in-depth research is urgently needed.

## 5. Conclusions and Perspectives

This article reviews recent research progress on the migration of MNP within plants and their impacts on plants. It thoroughly illustrates the pronounced effects of MNP on plant germination, growth, development, and litter decomposition. Furthermore, there is ample evidence of the persistent effects of MNP on the physiological and biochemical characteristics of plants in the environment. Due to the continuous advancement and development of analytical techniques, the mechanisms underlying the physiological and biochemical effects of MNP on terrestrial plants are gradually becoming clearer. Nevertheless, there is still a need for further exploration in the following areas:

(1) In order to gain a more comprehensive understanding of the impact of MNP, it is necessary to expand the scope of research and investigate the effects of various types of MNP on different plant species. MNP have diverse sources and types, but current research often relies on primary MNP for indoor experiments, and most MNP are artificially produced. However, MNP in the environment mostly exist in the form of secondary MNP, with differences in their physicochemical properties and the environment they inhabit compared to those used in laboratory experiments. This fails to reflect their actual state in natural soils. In the future, it is essential to delve deeper into the actual effects of MNP with different sources and characteristics on soil plants in real environmental conditions. Additionally, while experiments have been conducted on a limited number of plant species at the individual level, there is a lack of research on their effects on plant community structure, diversity, and primary production changes.
(2) To reveal the toxicological mechanisms of MNP on plants, further in-depth research is required to elucidate their effects at the molecular and genetic levels. Current research primarily focuses on the physiological effects of MNP on plants, with limited understanding of their toxic effects at the molecular and genetic levels. Advanced research techniques such as high-throughput genomics can be employed to further uncover the toxic mechanisms of MNP at the molecular and genetic levels.
(3) MNP not only affect plants but also exert influences on microorganisms, and there is an interaction between all three of them. Since microorganisms share similarities with NP, such as small size and a large surface area, they can come into direct contact with the external environment. They are also widely distributed in the environment, abundant in number, and diverse in species. It is crucial to elucidate the intrinsic mechanisms of the interaction between MNP, plants, and microorganisms. However, current research on the impact of MNP on endophytic microbial communities in plants and phyllosphere microbial communities is relatively limited. Further in-depth research is needed to investigate the effects of MNP on the structure and function of these microbial communities, as well as the underlying mechanisms of interaction among the three.
(4) During the decomposition of litter, microorganisms can form biofilms on the surface of MNP, and MNP may potentially serve as “electron shuttles”, participating in microbial metabolism as electron sinks or sources. However, the mechanisms by which MNP mediate the extracellular electron transfer by microorganisms remain unclear. Furthermore, soils contain a significant amount of natural “electron shuttles”, such as humic substances, and research on the interaction between MNP and these natural “electron shuttles” is currently limited. A deeper understanding of the relationship between MNP and “electron shuttles”, as well as their impact on microbial decomposition activities, is crucial in addressing the challenges posed by plastic pollution.

## Figures and Tables

**Table 1:** Effects of MNP on plant photosynthesis.

Test Object	MNP			Exposure Time	Position	Effect	References
	Type	Size	Concentration				
cabbage	PS	5 μm 70 nm	10 mg/kg	2 d	leaf surface	Plant photosynthesis and growth are affected, with chlorophyll a being more susceptible to influence than chlorophyll b.	[37]
oilseed seedlings	PMMA	63.3 ± 17.9 nm	0.05 g/L 0.5 g/L	6 d	leaf surface; root system	PMMA, when exposed through the roots, enters the stems and roots of canola seeds, with a greater impact on root tip cells and chloroplasts compared to leaf surface exposure.	[38]
mung bean	-	57–229 μm	0.1% 1.1%	28 d	leaf surface	MNP alter the flavonoid content and photosynthetic factors, resulting in a reduction in the amount of light absorbed by the plants.	[39]
madder	PS	1 μm 12 μm	10, 50 and 100 mg/L	10 d	seed	MNP with both 1 μm and 12 μm significantly increase the chlorophyll content and fluorescence in all treatment groups.	[40]
oilseed rape	PE	0.15 mm	0, 18 and 36 g/kg	3 m	surrounding the seed	PE affects the synthesis of chlorophyll b, with the most significant impact on chlorophyll b content; it reduces the ability of leaves to capture and convert light energy.	[41]

Note: PS, Polystyrene; PMMA, Polymethyl methacrylate; PE, Polyethylene; d, day; m, month.

## Data Availability

All data have been included in the main text.
